# Vibrational Characteristics of a Foam-Filled Short Basalt Fiber Reinforced Epoxy Resin Composite Beetle Elytron Plate

**DOI:** 10.3390/ma15217748

**Published:** 2022-11-03

**Authors:** Jinxiang Chen, Shengchen Du, Chaochao He, Nanxing Zhu

**Affiliations:** Key Laboratory of Concrete and Prestressed Concrete Structures of the Ministry of Education, Southeast University, Nanjing 211189, China

**Keywords:** sandwich plates, beetle elytron plate, composite, vibrational property, shear

## Abstract

The vibrational properties and mechanism of a foam-filling short basalt fiber reinforced epoxy resin composite beetle elytron plate (EBEP_fc_) were studied by experiments and the finite element (FE) method in this paper. The experimental results showed that the natural frequencies of the first two modes of the EBEP_fc_ were very close to those of a foam-filling short basalt fiber reinforced epoxy resin composite honeycomb plate (HP_fc_), while the vibrational response of the EBEP_fc_ was weaker than that of the HP_fc_, and the damping ratio was improved; the improvement of the second mode was significant. Therefore, the EBEP_fc_ had a better vibration reduction performance and could directly replace the HP_fc_ in engineering applications. The FE results showed that foam filling enhanced the shear stiffness of the whole core structure, and had a more obvious effect on the shear stiffness of the HP_fc_. Meanwhile, it particularly reduced the shear force proportions and contributed to the protection of the skin and core skeleton. The mechanisms of the vibrational characteristics of these two types of sandwich plates were explored from the perspective of the equivalent cross-sectional area, shear stiffness, shear strain energy per unit volume and friction. These results provide a valuable reference for the promotion and application of EBEP_fc_ in the fields of vibration reduction and seismic resistance.

## 1. Introduction

Sandwich structures, especially honeycomb structures, have been applied in transportation [1], aerospace [2,3], building structure [4,5] and other fields due to their lightweight, high-strength mechanical properties, good thermal insulation and vibrational performance. With the development of modern industrial technology and the needs of engineering applications, there are more demanding requirements for the structural performance of sandwich plates with regard to compression resistance, impact resistance, vibration reduction and sound insulation in engineering applications. Therefore, there are many experimental [6,7], finite element (FE) [8], and equivalent theory [9,10,11,12,13] studies on the vibrational properties of sandwich plates. For example, in terms of the vibrational properties of foam-filling sandwich plates, Florence et al. [14] studied the influence of three kinds of fillings by energy absorption materials on the natural frequency and damping of sandwich structures, and the results showed that polyurethane foam demonstrated the best effect. Boucher et al. [15] found that the partial filling of a honeycomb plate could significantly improve damping without significantly increasing the structural mass, so that the structure had a good damping effect. Mozafari et al. [8] used a numerical method to explore the influence of foam fillings with different densities on the natural frequency and vibrational modes of honeycomb plates. Sadowski et al. [16,17] reported the equivalent elastic properties of hollow honeycomb and foam-filled honeycomb structures by using the displacement-based homogeneous FE method, and calculated the natural frequencies of foam-filled honeycomb structures based on a new three-dimensional numerical FE model. These researchers found that the natural frequency of the foam-filling honeycomb plate was clearly affected by the face sheet, core and foam material. Arunkumar et al. [18] derived a two-dimensional equivalent model of a foam-filling sandwich plate, and found that foam filling could effectively improve the static and free vibrational performance of the sandwich plate. Kao et al. [19] explored the dynamic stability of a foam-filling sandwich plate under cyclic loading, and proved that reducing the core height could improve its dynamic stability.

**Figure 1:**
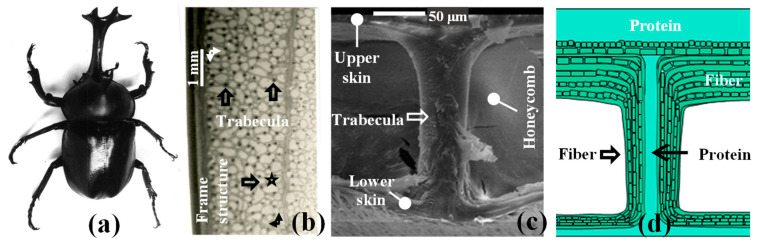
The origin of the BEP. (**a**) An adult A. dichotoma beetle; (**b**) microstructure of an A. dichotoma forewing; (**c**) trabeculae in the forewing; and (**d**) a simple model of a trabecular structure (Adapted from reference [32] with permission).

The core structural parameters also influenced the vibrational characteristics of the sandwich plates. Wang et al. [20] found that sandwich plate parameters such as face sheet thickness, honeycomb wall thickness and foam filling density had a significant influence on the natural frequency of the structure. Muhammet et al. [21] studied the influence of parameters on the natural frequency of the foam-filled honeycomb plates. These authors found that the damping ratio and natural frequency increased with increasing core height, and they used the Taguchi method to determine better structural parameters. Moreover, our research group was the first to propose the concept of a beetle elytron plate (BEP) [22]. The BEP is a new kind of bionic sandwich structure that was proposed in recent years. Characterized by a trabecular-honeycomb core structure (Figure 1d), the BEP is the result of our group’s research on the three-dimensional structure of the *Allomyrina dichotoma* beetle forewing (Figure 1a) since 1997. The influence of the dimensions and modelling methods of BEPs (with the same core thickness and the same core volume) on the mechanical properties of the BEPs has also been investigated [23,24]. The shared mechanism of trabeculae, the synergistic mechanism of the trabecular-honeycomb structure, and the excellent mechanical properties of EBEP have been recently investigated [25]. The results proved that the mechanical properties of compression [26], energy absorption [27], bending [28], and shearing [29] of the EBEP were significantly better than those of traditional honeycomb plates. In this context, the vibrational characteristics of a 3D-printed BEP composed of aluminum were proven in a previous study [30] to be better than those of a traditional honeycomb plate. The vibrational response of the BEP was approximately 25% less than that of the honeycomb plate, with almost the same first natural frequency. Recently, our research group investigated the compression and energy absorption characteristics of PVC foam-filling beetle elytron plates [31]. However, there have been no research studies regarding the vibrational characteristics of short basalt fiber-reinforced epoxy resin composite foam-filling beetle elytron plates. In particular, only the first natural frequency and its amplitude were investigated in a previous study, and the vibrational characteristics, such as the vibrational mode and damping ratio of the BEP, have not been studied.

Chanachai et al. explored the acousto-structural behavior of a sandwich cylindrical shell [33]; Özkan et al. conducted an experimental study on the impact resistance of glass fiber composite pipes [34]. These references, and the research of this group, have proven the excellent comprehensive performance of the thin-walled structure of composite sandwich panels. The short basalt fiber-reinforced epoxy resin composites have excellent properties, such as a light weight, high strength, impact resistance and corrosion resistance [35]. In this study, the natural frequency, damping ratio, vibrational mode and vibrational response of a foam-filling short basalt fiber-reinforced epoxy resin composite honeycomb plate (HP_fc_) and beetle elytron plate (EBEP_fc_) were determined via modal experiments. The influence mechanism of foam filling on the shear characteristics of the HP_fc_ and EBEP_fc_ were revealed through finite element analysis (FEA). The vibrational superiority of EBEP_fc_ was clarified from the perspective of shear modulus and friction. The results of this research lay a good experimental foundation for promoting the engineering application of foam-filling BEPs.

## 2. Sample Preparation and Experimental Method

### 2.1. Sample Design and Preparation

**Figure 2:**
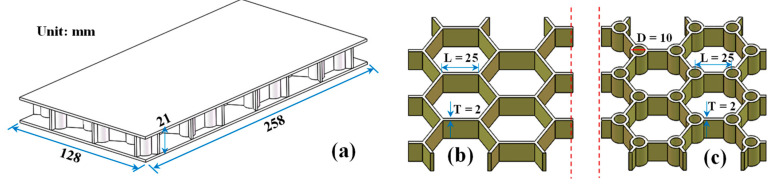
Structural size diagram of the sandwich plate. (**a**) Overall size (EBEP_fc_ as an example); (**b**) EBEP_fc_ core unit size; and (**c**) HP_fc_ core unit size. No filling foam was displayed here.

The overall dimensions of the HP_fc_ and EBEP_fc_ are shown in Figure 2. The dimensions of the two plates were 258 mm × 128 mm × 21 mm; the upper and lower face sheet thicknesses were T_f_ = 2 mm, the core height was h = 17 mm, the honeycomb wall thickness was T = 2 mm, and the center radius of the trabeculae was R = 5 mm. The preparation processes of the HP_fc_ and EBEP_fc_ were introduced in detail in the literature [36]. The volume ratio of glue:basalt fiber was 3:1, and the mass ratio of the glue was resin:curing agent:diluent = 10:3:1. The density of the basalt fiber was 2650 kg/m^3^, the density of the PVC foam was 330 kg/m^3^, its compressive strength was 2 MPa, its elastic modulus was 47 MPa, and its Poisson’s ratio was 0.32.

### 2.2. Modal Experiment

**Figure 3:**
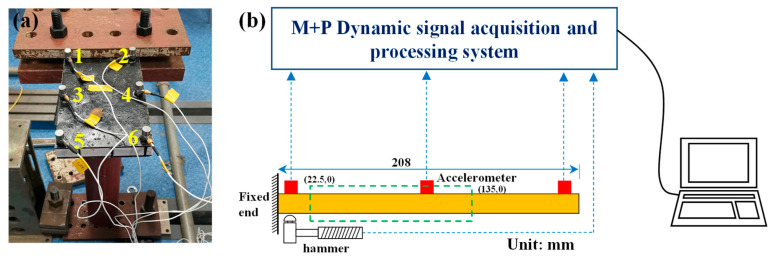
Schematic diagram of the modal experiment. (**a**) Clamped sample and measuring points (marked with number from 1 to 6) arrangement, and (**b**) schematic diagram of the experimental device.

The experiment was conducted at room temperature at the Institute of Structural Vibration, Nanjing University of Aeronautics and Astronautics (Nanjing, China). The boundary condition was one end clamped and one end free (Figure 3a,b). The clamped end was fixed by bolts, and the base was fixed on the ground to ensure sufficient stiffness. The length of the clamped end was 50 mm, and the cantilever length was 208 mm. The natural frequencies of the two sandwich plates were obtained by the force hammer method. Six measuring points were arranged at the base, middle and free end of the cantilever, as shown in Figure 3a, and PCB piezoelectric sensors (PCB 352C33; PCB Co., Ltd., Greenbrae, CA, USA; sensitivity: 100.1 mV/N) were attached by glue. At the cantilever root measuring point 1, the excitation was input by a force hammer (NTS-LW-141-110; M+P Company, Hannover, Germany; sensitivity: 2.5 mV/N). Each sample was tested three times to confirm that the experiment was not affected by noise (the results of three experiments were very close), and then one of them was selected as the experimental result. The corresponding response signal was obtained by the PCB piezoelectric acceleration sensor, and the frequency response curve and related modal parameters of the two sandwich plates were obtained by a dynamic signal acquisition and processing system (VMAI810, M+P Company, Hannover, Germany) through Fourier transform. The damping ratio of the structure was obtained by the half-power bandwidth method.

**Figure 4:**
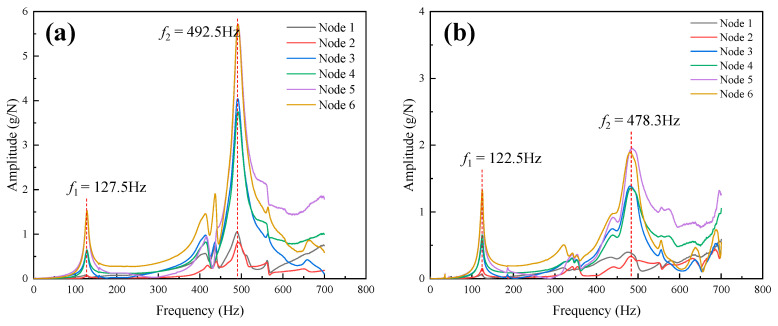
Experimental frequency response curves of two types of sandwich plates: (**a**) HP_fc_ and (**b**) EBEP_fc_.

Considering the measured first-order and second-order natural frequencies (see Figure 4 below for specific values), the peak response of the first- and second-order natural frequencies *f*_0_ was determined in the frequency response curve, and the frequencies *f*_1_ and *f*_2_ corresponding to 0.707 times the response peak were found. Then, the damping ratio *ξ* was calculated by the following Equation (1):

(1) $$\xi =\frac{f_{2}-f_{1}}{2f_{0}}$$

### 2.3. FE Simulation of Two Sandwich Plates under a Transverse Load

To investigate the vibrational characteristics of the EBEP_fc_, in this study, the difference in the vibrational characteristics of the HP_fc_ and EBEP_fc_ were explored from the shear force proportion characteristics under a transverse static load, by using the method applied in our previous research [30]. Notably, although the inertial force caused by mass difference leads to differences in the dynamic and static mechanical properties of sandwich plates under transverse loading, when the structure is within the elastic range, its dynamic and static responses are usually positively correlated [30,32]. Therefore, a FE static loading simulation was used to explore the shear characteristics and the vibrational performance of the HP_fc_ and EBEP_fc_.

The sandwich plate and foam model in Figure 2 were drawn by SolidWorks 2016 (Dassault Systèmes, Vélizy-Villacoublay, France), and were imported into ABAQUS for assembly. C3D8R solid elements were used to simulate the material properties (Table 1 measured in a previous paper [36]). After the initial analysis, the mesh feature size was determined to be 2 mm. The tie constraint was adopted between the foam and the fiber composite. The boundary conditions were one end fixed and one end free. In view of the periodic structure of the two sandwich plates along the length direction, when exploring the shear force proportion characteristics of the HP_fc_ and EBEP_fc_, a concentrated static load (10 N) was applied at the cantilever end, and the relevant data were extracted in the post-processing step. The distributions of the shear forces of the EBEP_fc_ and HP_fc_ were investigated by selecting the slightly off-base section in the middle of the cantilever (Figure 3b, virtual line box: coordinates (22.50) to (135.0)). The influence mechanism on the vibrational characteristics of the above two sandwich plates were also explored.

## 3. Results and Discussion

### 3.1. Results and Analysis of the Vibration Experiment of the HP_fc_ and EBEP_fc_

**Figure 5:**
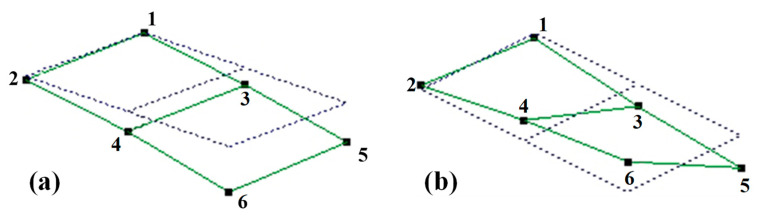
The first two vibrational modes of two kinds of sandwich plates obtained by the M+P dynamic analysis system (measuring points are marked with number from 1 to 6): (**a**) first-order vibrational mode (first-order bending) and (**b**) second-order vibrational mode (first-order torsion).

**Figure 6:**
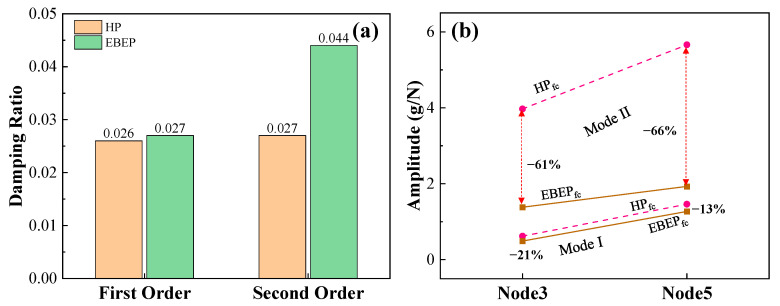
The vibrational characteristics of the two kinds of sandwich plates for the first two vibrational modes: (**a**) damping ratio and (**b**) the first two vibrational amplitude responses of different nodes.

Figure 4 shows the frequency response curves of the HP_fc_ and EBEP_fc_; the ordinate was the acceleration response (hereafter referred to as the amplitude response). The natural frequency of the EBEP_fc_ was slightly lower than that of the HP_fc_. The first-order natural frequency of the HP_fc_ was 127.5 Hz, and the second-order natural frequency was 492.5 Hz. The first-order natural frequency of the EBEP_fc_ was 122.5 Hz, which was 3.9% lower than that of HP_fc_, and the second-order natural frequency was 478.3 Hz, which was 2.9% lower than that of HP_fc_. Compared with a previous study [30], the first-order natural frequency of a 3D-printed aluminum alloy HP was approximately 2% lower than that of an EBEP. Although all the differences were small in both studies, the natural frequency of the EBEP_fc_ in this study decreased more from the first-order to the second-order mode. Concerning the first two orders of the modes of the two sandwich plates, the results obtained by the post-processing program of the data analysis software in the experiment are shown in Figure 5. The first two modes of the HP_fc_ and EBEP_fc_ were the same. That is, the first-order mode was the first-order bending mode, and the second-order mode was the first-order torsional mode. Then, we investigated the damping ratio of the first two modes of the HP_fc_ and EBEP_fc_ (Figure 6a). Figure 6 shows that in terms of the first-order mode, the damping ratio of the EBEP_fc_ was similar to that of the HP_fc_, but the second-order damping ratio was significantly increased by 83% compared with that of the HP_fc_. This characteristic can effectively reduce the amplitude response of the EBEP_fc_ in the torsional mode. In the bending mode, the energy was mainly dissipated by the tensile and compressive deformation of the core, while in the torsional mode, the energy was generally dissipated by the shear deformation of the core. Shear deformation is more effective than tension–compression for the dissipation of the energy in a structure [37]. Shear deformation may involve higher modal strain energy dissipation, which will be investigated in detail in future research.

When the frequency of the external dynamic load is close to the natural frequency of a structure, the vibrational response of the structure is extremely large, which reduces the safety and comfort of the structure. In this study, the natural frequencies of the HP_fc_ and EBEP_fc_ were quite close, so the vibrational performance of the HP_fc_ and EBEP_fc_ were evaluated by exploring their vibrational responses. Therefore, Figure 6b gives the maximum amplitude response corresponding to the first two natural frequencies of the middle and end of the cantilevers of the HP_fc_ and EBEP_fc_. Figure 6b shows that for the first-order vibrational mode, the amplitude responses of the EBEP_fc_ at the middle and end nodes of the cantilever were 21% and 13% lower than that of the HP_fc_, while the second-order vibrational modes were 61% and 66% lower, respectively. Therefore, even when filled with foam, the EBEP_fc_ could effectively reduce the vibrational amplitude response without basically changing the natural vibrational frequency, so it had a good vibration reduction effect. Thus, this phenomenon provided an experimental basis for using the EBEP_fc_ to replace the HP_fc_ to improve the overall vibrational performance and vibration comfort of the structure under similar working conditions. That is, EBEP_fc_ had a better damping performance. For the first-order mode, the amplitude response rise trends of different nodes of the HP_fc_ and EBEP_fc_ were relatively consistent, which was also consistent with the result that their first-order damping ratios were very close. The EBEP_fc_ has a better damping performance. As mentioned in the Introduction, the EBEP had far better mechanical properties, such as compression, energy absorption, bending and shearing, than the HP. The foam filling and integrated preparation method adopted in this study greatly improved the performance of the EBEP in terms of energy savings, heat preservation and sound insulation, and the mechanical properties were better than even multibody molding. Thus, the EBEP filled with foam and prepared in an integrated way is expected to be used in the fields of high-speed railways, marine transportation, aerospace, and buildings, with demanding requirements for light weight and insulation. The EBEP can also be used as an excellent substitute for the HP.

The next section discusses the use of the FE method to explore the difference in vibrational characteristics between the two kinds of sandwich plates from the shear stress characteristics of the HP_fc_ and EBEP_fc_ under transverse static loading.

### 3.2. FE Analysis Results and Discussion

Since the displacement of the cantilever sandwich plate under transverse static load are determined by its bending and shear properties, and the bending performance and mechanism of the BEP have been discussed in detail [38], the vibrational characteristics of the EBEP_fc_ and HP_fc_ are further compared and revealed from the shear characteristics of the HP_fc_ and EBEP_fc_ under a transverse static load.

#### 3.2.1. FE Analysis Results

**Figure 7:**
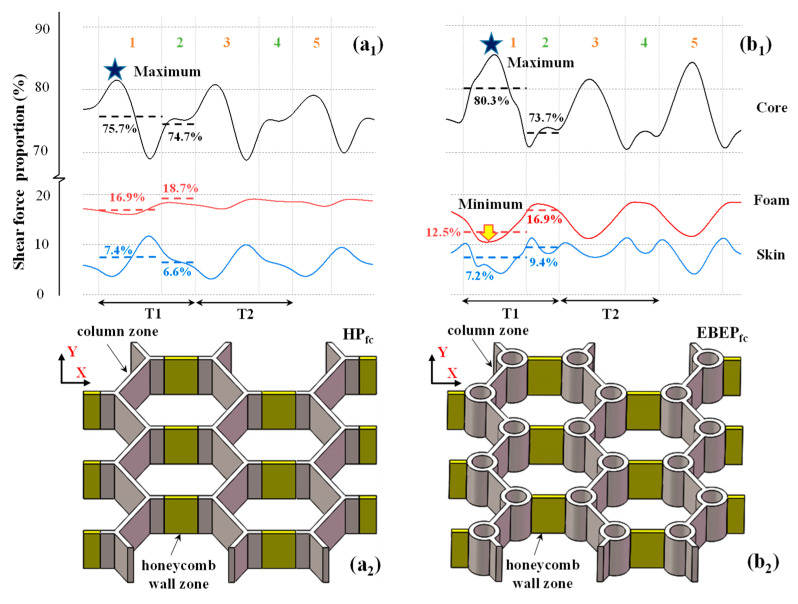
The core structure in the calculation interval and the FE results of the shear force proportions of the BFRP core skeleton, foam and skin of the two sandwich plates: (**a_1_**,**a_2_**) HP_fc_ and (**b_1_**,**b_2_**) EBEP_fc_. The shear force proportion of the skin refers to the ratio of the sum of the shear forces born by the upper and lower skins to the total shear force. The data in the figure are the average values in the calculation intervals. The numbers in (**a_1_**,**b_1_**) show column zones (orange numbers) and honeycomb wall zones (green numbers).

**Figure 8:**
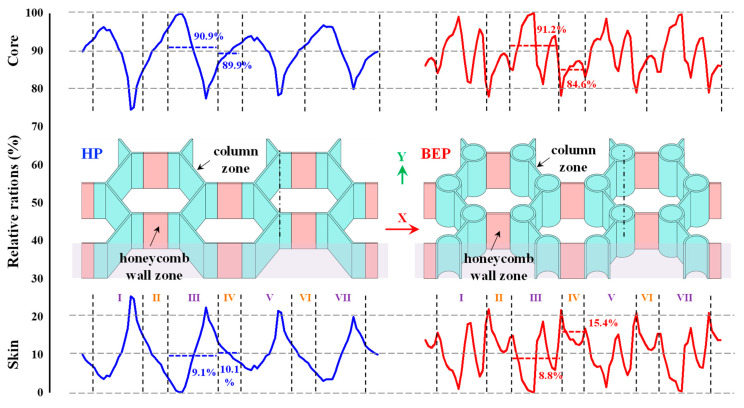
The FE results of the shear force proportions of the core skeleton and skin (unfilled metal HP and BEP (η = 0.25)) (Adapted from reference [30] with permission). Upper and lower rows (blue and red curves): proportions of the shear force in the core structure and skins of the honeycomb plate and BEP. For convenience of observation, the skins are not shown, and the half-structure of the core structure along the Y-direction is displayed. The Roman numerals show column zones (purple) and honeycomb wall zones (orange).

First, the shear force proportions of the two kinds of sandwich plates filled with foam were investigated. Thus, Figure 7 shows the shear force proportions of the basalt fiber-reinforced polymer (BFRP) core skeleton, foam, and skin of the HP_fc_ and EBEP_fc_ in the virtual line box in Figure 3b. Since the shear force distribution was repeated in a cycle (T) of 37.5 mm along the X-direction (Figure 7), the method used in a previous study [30] was applied here; the structure of a sandwich plate in a cycle was divided into two areas, namely, the trabeculae area and honeycomb wall area (Figure 7a_2_,b_2_). The honeycomb was regarded as an open triangular column with a diameter of 0 [30]. For convenience of comparison, Figure 8 also cited the results of the unfilled metal HP and BEP [30]. A comparison of Figure 7 and Figure 8 shows that after foam filling, the foam in the HP_fc_ and EBEP_fc_ bore averages of 16.9% and 12.5% of the shear force in the trabeculae area, and 18.7% and 16.9% of the shear force in the honeycomb wall area, respectively. The foam significantly reduced the average shear force proportion of the core skeleton and skin of the two sandwich plates, and the fluctuation range decreased. In particular, when there was no foam filling, the maximum shear force proportion of the core in the trabeculae area of the two sandwich plates reached 100% (Figure 8). In this study, after foam filling, the proportions decreased to 81% (HP_fc_) and 85% (EBEP_fc_). The maximum shear force proportion of the skin of HP_fc_ decreased from 26% (before filling) to 12% (after filling), and that of EBEP_fc_ decreased from 23% (before filling) to 10% (after filling). Although the foam was weak, not only the shear force proportion of the overall core structure (core skeleton + foam), but also the overall shear force proportion of the core increased after filling. Additionally, the proportion of the shear force borne by the foam filling exceeded that of the skin of the sandwich plate, which played a positive role in protecting the core skeleton and the skin. It also enhanced the shear stiffness of the two sandwich plates to a certain extent.

Second, the difference between the HP_fc_ and EBEP_fc_ after foam filling was investigated. As mentioned above, in the trabeculae area, the average shear force proportion of foam in the HP_fc_ was 16.9%, which was greater than that of the EBEP_fc_ (12.5%, Figure 7a_1_,b_1_). However, the highest shear force proportion of the BFRP core skeleton of EBEP_fc_ was also greater than that of the HP_fc_ (Figure 7a_1_,b_1_, asterisk). This clearly was because the trabeculae gave full play to their structural advantage of their sharing mechanism [22]. This enhanced the core skeleton so that it bore more shear force in the synergy between the trabeculae structure and foam. Accordingly, the shear force proportion of the EBEP_fc_ foam decreased (the trough indicated by the downwards wide arrow in Figure 7b_1_). Compared with the situation in the EBEP_fc_, the foam had a stronger reinforcing effect on the core skeleton of the HP_fc_. This was consistent with the conclusion that the foam filling could enhance the mechanical properties of weaker structures. From the above discussion, it was clear that the foam filling could enhance the shear stiffness of the HP_fc_ and EBEP_fc_ cores, which also improved their static and dynamic properties to a certain extent.

#### 3.2.2. Internal Mechanism Influencing the Vibrational Characteristics of the EBEP_fc_

The superior vibrational acceleration response and the damping ratio of the internal mechanism of the EBEP_fc_ relative to those of the HP_fc_ were investigated from the perspective of the shear modulus characteristics of the core and friction.

**Figure 9:**
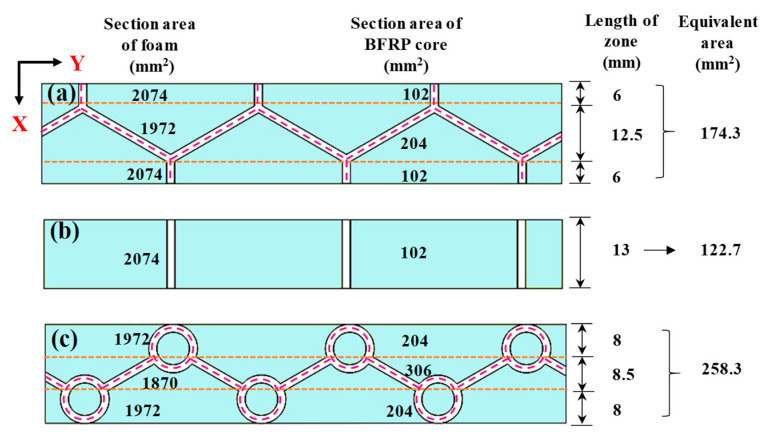
Diagram of the analysis of the equivalent cross-sectional area of the trabeculae and honeycomb wall of the HP_fc_ and EBEP_fc_: (**a**) trabeculae area of the HP_fc_; (**b**) honeycomb wall area of the HP_fc_ and EBEP_fc_; and (**c**) trabeculae area of the EBEP_fc_. The data in the first and second columns from the left in the figure are the cross-sectional areas of each foam and BFRP core skeleton, respectively.

According to the sandwich plate theory [39], in the elastic stage, the shear stiffness C of the core is directly proportional to the shear modulus G_c_ of the core structure, that is, C = G_c_h. In this study, the shear modulus of each section was different due to the presence of a honeycomb, a BEP unit and foam. To simplify the calculation and analysis, as in the literature [30], the equivalent cross-sectional area of the trabeculae area and honeycomb wall area were calculated to qualitatively reflect the change in the shear modulus. Considering that the PVC foam accounted for a large proportion of the cross-sectional area, the PVC foam was equivalent to the BFRP according to its stiffness to simplify the analysis; thus, 1% of the PVC cross-sectional area was converted into the equivalent cross-sectional area. Due to the complex structure of the honeycomb wall cross section, the calculation of the cross-sectional area was cumbersome. In this paper, the following simplified method was adopted when calculating the equivalent cross-sectional area: any equivalent cross-sectional area = the number of honeycomb walls in the cross section × honeycomb wall thickness (2 mm) × core height (17 mm) + foam sectional area/100. Using this simplification, the equivalent cross-sectional area of each region was calculated by weighted averaging according to the length and equivalent cross-sectional area of each part, to approximately evaluate the change in the equivalent shear modulus of the core. The results for the equivalent cross section of each area are shown in Figure 9 (the data in the rightmost column of Figure 9).

Figure 9 shows that in the trabeculae area, because of the presence of trabeculae, the sum of the honeycomb wall area in its cross section was large. That is, the equivalent cross-sectional area of the EBEP_fc_ was 258.3, while that of the HP_fc_ was 174.3, and the equivalent cross-sectional area of the EBEP was 48.5% higher than that of the HP. The shear modulus and shear stiffness of the EBEP_fc_ in the trabeculae area were significantly higher than those of the HP_fc_. The dynamic and static responses of the structure within the elastic stage were directly proportional [30]. Therefore, it was inferred that the EBEP_fc_ had greater shear stiffness, which made its dynamic and static responses significantly smaller than those of the HP_fc_. However, the EBEP_fc_ had a higher shear modulus than the HP_fc_, which caused the sandwich plate to undergo a smaller shear deformation under the same energy input, which resulted in a smaller shear strain energy per unit volume. According to the research of Gounaris et al. [40], the damping ratio was inversely proportional to the shear strain energy per unit volume. Therefore, a higher shear modulus in the trabeculae area of the EBEP brought a larger damping ratio and more effective energy dissipation, which allowed for a better vibrational response performance than in the case of the HP_fc_. This was verified by the observation that the damping ratio of EBEP_fc_ was greater than that of the HP_fc_ in the previous experiment.

Finally, from the perspective of friction, this study explained another reason that the second-order damping ratio of the EBEP_fc_ was 83% higher than that of the HP_fc_. In the transverse vibration process of the core layer, energy was dissipated by the interfacial friction between the foam and the core skeleton of the fiber composite from a macroscopic point of view, and the friction between the short fibers in the core skeleton, the friction between the short fibers and the resin matrix, and the friction between the epoxy resin molecules from a microscopic perspective. The microscale reasons might be dominant, and the damping ratio would be significantly affected by these factors. According to Figure 7, the peak shear force proportion of the core skeleton of the EBEP_fc_ was higher than that of the HP_fc_. Therefore, it was inferred that in the dynamic response, the stress amplitude in the EBEP_fc_ was also larger. The energy consumption per unit volume of material increased with the increasing stress amplitude, which led to more intense friction in the fiber composite skeleton and thus dissipated more energy. Therefore, the damping ratio of the EBEP_fc_ was also higher than that of the HP_fc_. The above discussion qualitatively explained why the first- and second-order damping ratios of the EBEP_fc_ were higher than those of the HP_fc_, which led to more energy dissipation by EBEP_fc_ in the transverse vibrational mode. Therefore, the vibrational response of the EBEP_fc_ was less than that of HP_fc_.

## 4. Conclusions

In this study, the modal parameters and vibrational responses of two foam-filled sandwich plates, HP_fc_ and EBEP_fc_, were obtained through modal experiments, their damping ratios under corresponding vibrational modes were calculated by the half-power bandwidth method, and their shear characteristics were explored through FE simulation. The differences between the vibrational characteristics of the HP_fc_ and EBEP_fc_ were analyzed from the perspective of shear modulus and friction, and the following conclusions were obtained:

(1) The first and second natural frequencies of the EBEP_fc_ were lower than those of the HP_fc_, but they were very close, and the first two modes were the same. The first- and second-order damping ratios of the EBEP_fc_ were greater than those of the HP_fc_, and the vibrational response was significantly less than that of the HP_fc_. Therefore, the EBEP_fc_ not only had a better vibration damping performance than HP_fc_ but could also directly replace the HP_fc_ to improve the vibration damping and seismic performance in practical engineering applications.
(2) Although the foam was weak, it reduced the shear force proportions of the core skeleton and the skin and its fluctuation range in the cycle. It enhanced the shear stiffness of the overall core structure, and further improved their static and dynamic performance. The foam played a positive role in protecting the core skeleton and skin. Additionally, the foam had a greater enhancement effect on the HP_fc_ because the trabeculae inside the EBEP_fc_ could bear more shear load, that is, the synergy between the trabeculae structure and foam bore more shear force.
(3) The vibrational performance of EBEP_fc_ was better than that of the HP_fc_, because the equivalent cross-sectional area of the trabeculae area of the core was greater than that of the HP_fc_. Thus, the shear modulus, shear stiffness and shear force proportion of the core skeleton were greater than those of the HP_fc_. As a result, the EBEP_fc_ had a better static and dynamic performance, a lower shear strain energy per unit volume and a greater damping ratio than the HP_fc_. Additionally, the EBEP_fc_ exhibited greater stress amplitude fluctuation in the dynamic response. This resulted in more intense friction, more energy dissipation and a greater damping ratio in the material, which also led to a better vibrational performance of the EBEP_fc_. The results of this study provide useful guidance for further research and applications of the EBEP_fc_ in the future.

## Figures and Tables

**Table 1:** Material properties for the FEA (Data from [36]).

	Density (kg/m^3^)	Compressive Strength (MPa)	Elastic Modulus (MPa)	Poisson’s Ratio
BFRP	1550	111.9	3693	0.3
PVC	330	2	47	0.32

## Data Availability

All the data is available within the manuscript.
